# 11-[(*E*)-Benzyl­idene]-14-hy­droxy-8-phenyl-3,13-diaza­hepta­cyclo­[13.7.1.1^9,13^.0^2,9^.0^2,14^.0^3,7^.0^19,23^]tetra­cosa-1(22),15,17,19(23),20-pentaen-10-one

**DOI:** 10.1107/S1600536810043874

**Published:** 2010-11-06

**Authors:** Raju Suresh Kumar, Hasnah Osman, Aisyah Saad Abdul Rahim, Madhukar Hemamalini, Hoong-Kun Fun

**Affiliations:** a School of Chemical Sciences, Universiti Sains Malaysia, 11800 USM, Penang, Malaysia; b School of Pharmaceutical Sciences, Universiti Sains Malaysia, 11800 USM, Penang, Malaysia; c X-ray Crystallography Unit, School of Physics, Universiti Sains Malaysia, 11800 USM, Penang, Malaysia

## Abstract

In the title compound, C_35_H_30_N_2_O_2_, the piperidine ring adopts a chair conformation and the pyrrolidine ring adopts an envelope conformation. The naphthalene ring makes dihedral angles of 24.56 (3) and 36.13 (4)° with the terminal phenyl rings. The dihedral angle between the two terminal phenyl rings is 55.27 (5)°. One of the C atoms in the pyrrolidine ring is disordered over two sites, with a refined occupany ratio of 0.670 (3):0.330 (3). An intra­molecular O—H⋯N hydrogen bond generates an *S*(6) ring. In the crystal structure, inversion dimers linked by pairs of C—H⋯O hydrogen bonds generate *R*_2_^2^(18) loops within sheets of mol­ecules lying parallel to the *bc* plane.

## Related literature

For the details of cyclo­addition reactions, see: Padwa (1984 ▶); Grigg & Sridharan (1993 ▶); Monlineux (1987 ▶). For puckering parameters, see: Cremer & Pople (1975 ▶). For the stability of the temperature controller used in the data collection, see: Cosier & Glazer (1986 ▶).
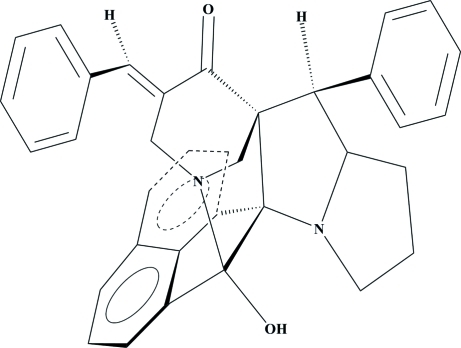


## Experimental

### 

#### Crystal data

C_35_H_30_N_2_O_2_
*M* *_r_* = 510.61
Monoclinic, 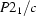

*a* = 11.2264 (19) Å
*b* = 15.600 (3) Å
*c* = 15.031 (3) Å
β = 93.927 (5)°
*V* = 2626.2 (8) Å^3^
*Z* = 4
Mo *K*α radiation
μ = 0.08 mm^−1^
*T* = 100 K
0.50 × 0.39 × 0.12 mm

#### Data collection

Bruker APEXII DUO CCD diffractometer
Absorption correction: multi-scan (*SADABS*; Bruker, 2009 ▶) *T*_min_ = 0.961, *T*_max_ = 0.991
44214 measured reflections
11860 independent reflections
9771 reflections with *I* > 2σ(*I*)
*R*_int_ = 0.027

#### Refinement

*R*[*F* ^2^ > 2σ(*F* ^2^)] = 0.046
*wR*(*F* ^2^) = 0.145
*S* = 1.10
11860 reflections
366 parameters
H atoms treated by a mixture of independent and constrained refinement
Δρ_max_ = 0.56 e Å^−3^
Δρ_min_ = −0.43 e Å^−3^

### 

Data collection: *APEX2* (Bruker, 2009 ▶); cell refinement: *SAINT* (Bruker, 2009 ▶); data reduction: *SAINT*; program(s) used to solve structure: *SHELXTL* (Sheldrick, 2008 ▶); program(s) used to refine structure: *SHELXTL*; molecular graphics: *SHELXTL*; software used to prepare material for publication: *SHELXTL* and *PLATON* (Spek, 2009 ▶).

## Supplementary Material

Crystal structure: contains datablocks global, I. DOI: 10.1107/S1600536810043874/hb5701sup1.cif

Structure factors: contains datablocks I. DOI: 10.1107/S1600536810043874/hb5701Isup2.hkl

Additional supplementary materials:  crystallographic information; 3D view; checkCIF report

## Figures and Tables

**Table 1:** Hydrogen-bond geometry (Å, °)

*D*—H⋯*A*	*D*—H	H⋯*A*	*D*⋯*A*	*D*—H⋯*A*
O2—H1O2⋯N2	0.877 (18)	1.942 (18)	2.6134 (11)	132.2 (15)
C35—H35*A*⋯O2^i^	0.93	2.54	3.3159 (13)	142

Symmetry code: (i) 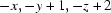.

**Fig. 1.**
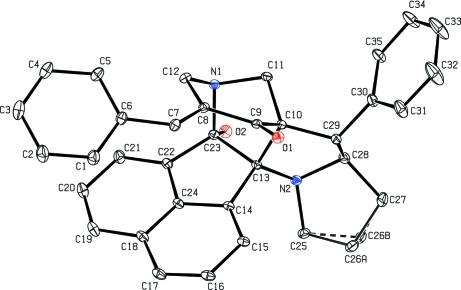
The asymmetric unit of the title compound, showing 30% probability displacement ellipsoids (H atoms are omitted for clarity). Dotted lines represents the disorder component.

**Fig. 2.**
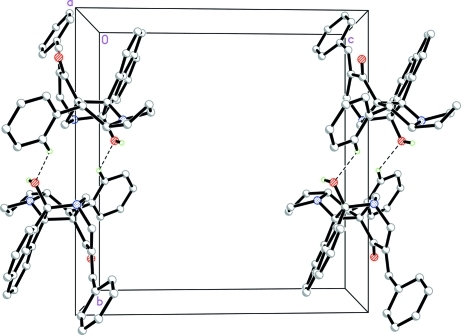
The crystal packing of the title compound, showing hydrogen-bonded (dashed lines) dimers. H atoms are not involving the hydrogen bond interactions are omitted for clarity.

## supplementary crystallographic information

### Comment

The intermolecular [3+2]-cycloaddition of azomethine ylides with olefinic dipolarophiles affords a number of novel heterocyclic scaffolds which are useful for the creation of diverse chemical libraries of drug-like molecules for biological screening (Padwa, 1984; Grigg & Sridharan, 1993). Functionalized pyrrolizidines are the central skeleton for numerous alkaloids and constitute classes of compounds with significant biological activity (Monlineux, 1987). In view of the biological significance of pyrrolizidines, the crystal structure determination of the title compound was carried out and the results are presented here.

The molecular structure of the title compound is shown in Fig. 1. The piperidine (N1/C8–C12) ring adopts a chair conformation [Q = 0.6060 (8) Å, θ = 141.15 (8)°, φ = 236.37 (12)°; Cremer & Pople, 1975]. The pyrrolidine ring, one of the C atom (C26) disordered over two sites with a refined occupancy ratio of 0.670 (3):0.330 (3). The major (N2/C25/C26A/C27/C28) and minor (N2/C25/C26B/C27/C28) disordered pyrrolidine rings adopt the same conformation, that is the envelope conformation; puckering parameters  Q(2) = 0.3547 (12) Å, φ = 257.52 (15)° for major disordered component and Q(2) = 0.3237 (17) Å, φ =  67.7 (2)° for minor disordered component. The naphthalene (C14–22/24) ring makes dihedral angles of 24.56 (3)° and 36.13 4)° with the terminal phenyl (C1–C6)/(C30–C35) rings. The dihedral angle between the two terminal phenyl rings (C1–C6) and (C30–C35) is 55.27 (5)°.

In the crystal packing (Fig. 2), adjacent molecules are connected by intramolecular O2—H1O2···N2 and intermolecular C35—H35A···O2 (Table 1) hydrogen bonds, forming dimers lying on sheets parallel to the *bc* plane.

### Experimental

A mixture of 3,5-bis[(*E*)-phenylmethylidene]tetrahydro-4(1*H*)-pyridinone (0.100 g, 0.363 mmol), acenaphthenequinone (0.066 g, 0.363 mmol) and proline (0.042 g, 0.363 mmol) were dissolved in methanol (5 ml) and refluxed for 30 min. After completion of the reaction as evident from TLC, the mixture was poured into water (50 ml). The precipitated solid was filtered and washed with water to afford the product which was recrystallised from ethyl acetate to reveal the title compound as colourless plates.

### Refinement

The hydroxyl H atom H1O2 was located from a difference Fourier map and refined freely. The remaining H atoms were positioned geometrically [C—H = 0.93–0.97 Å] and were refined using a riding model, with *U*_iso_(H) = 1.2*U*_eq_(C). One of the C atom (C26) of the pyrrolidine ring and the associated H atoms H25A, H25B, H26A, H26B, H27A and H27B disordered over two sites with a refined occupancy ratio of 0.670 (3):0.330 (3).

### Crystal data

**Table:**

C_35_H_30_N_2_O_2_	*F*(000) = 1080
*M**_r_* = 510.61	*D*_x_ = 1.291 Mg m^−^^3^
Monoclinic, *P*2_1_/*c*	Mo *K*α radiation, λ = 0.71073 Å
Hall symbol: -P 2ybc	Cell parameters from 9930 reflections
*a* = 11.2264 (19) Å	θ = 2.7–35.4°
*b* = 15.600 (3) Å	µ = 0.08 mm^−^^1^
*c* = 15.031 (3) Å	*T* = 100 K
β = 93.927 (5)°	Plate, colourless
*V* = 2626.2 (8) Å^3^	0.50 × 0.39 × 0.12 mm
*Z* = 4	

### Data collection

**Table:**

Bruker APEXII DUO CCD diffractometer	11860 independent reflections
Radiation source: fine-focus sealed tube	9771 reflections with *I* > 2σ(*I*)
graphite	*R*_int_ = 0.027
φ and ω scans	θ_max_ = 35.4°, θ_min_ = 1.9°
Absorption correction: multi-scan (*SADABS*; Bruker, 2009)	*h* = −18→17
*T*_min_ = 0.961, *T*_max_ = 0.991	*k* = −25→25
44214 measured reflections	*l* = −24→24

### Refinement

**Table:**

Refinement on *F*^2^	Primary atom site location: structure-invariant direct methods
Least-squares matrix: full	Secondary atom site location: difference Fourier map
*R*[*F*^2^ > 2σ(*F*^2^)] = 0.046	Hydrogen site location: inferred from neighbouring sites
*wR*(*F*^2^) = 0.145	H atoms treated by a mixture of independent and constrained refinement
*S* = 1.10	*w* = 1/[σ^2^(*F*_o_^2^) + (0.0795*P*)^2^ + 0.4943*P*] where *P* = (*F*_o_^2^ + 2*F*_c_^2^)/3
11860 reflections	(Δ/σ)_max_ = 0.001
366 parameters	Δρ_max_ = 0.56 e Å^−^^3^
0 restraints	Δρ_min_ = −0.43 e Å^−^^3^

### Special details

**Table:**

Experimental. The crystal was placed in the cold stream of an Oxford Cryosystems Cobra open-flow nitrogen cryostat (Cosier & Glazer, 1986) operating at 100.0 (1) K.
Geometry. All s.u.'s (except the s.u. in the dihedral angle between two l.s. planes) are estimated using the full covariance matrix. The cell s.u.'s are taken into account individually in the estimation of s.u.'s in distances, angles and torsion angles; correlations between s.u.'s in cell parameters are only used when they are defined by crystal symmetry. An approximate (isotropic) treatment of cell s.u.'s is used for estimating s.u.'s involving l.s. planes.
Refinement. Refinement of F^2^ against ALL reflections. The weighted R-factor wR and goodness of fit S are based on F^2^, conventional R-factors R are based on F, with F set to zero for negative F^2^. The threshold expression of F^2^ > 2σ(F^2^) is used only for calculating R-factors(gt) etc. and is not relevant to the choice of reflections for refinement. R-factors based on F^2^ are statistically about twice as large as those based on F, and R- factors based on ALL data will be even larger.

### Fractional atomic coordinates and isotropic or equivalent isotropic displacement parameters (Å^2^)

**Table:**

	*x*	*y*	*z*	*U*_iso_*/*U*_eq_	Occ. (<1)
O1	−0.17017 (5)	0.82679 (4)	1.03805 (4)	0.01641 (11)	
O2	0.08100 (6)	0.56861 (4)	0.87384 (4)	0.01734 (11)	
N1	0.08145 (6)	0.63805 (4)	1.01254 (4)	0.01357 (11)	
N2	−0.13097 (6)	0.63195 (4)	0.83198 (4)	0.01472 (12)	
C1	0.22693 (8)	0.98132 (6)	1.04040 (7)	0.02372 (17)	
H1A	0.1715	1.0100	1.0024	0.028*	
C2	0.34217 (9)	1.01357 (7)	1.05405 (8)	0.0296 (2)	
H2A	0.3638	1.0632	1.0249	0.035*	
C3	0.42518 (9)	0.97128 (8)	1.11159 (8)	0.0305 (2)	
H3A	0.5029	0.9919	1.1199	0.037*	
C4	0.39160 (9)	0.89812 (7)	1.15656 (7)	0.02777 (19)	
H4A	0.4465	0.8707	1.1960	0.033*	
C5	0.27640 (8)	0.86573 (6)	1.14284 (6)	0.02156 (16)	
H5A	0.2545	0.8170	1.1734	0.026*	
C6	0.19310 (7)	0.90631 (5)	1.08312 (5)	0.01729 (14)	
C7	0.07160 (7)	0.87336 (5)	1.06380 (5)	0.01624 (13)	
H7A	0.0115	0.9142	1.0555	0.019*	
C8	0.03709 (7)	0.79064 (5)	1.05664 (5)	0.01353 (12)	
C9	−0.09132 (7)	0.77452 (5)	1.02769 (4)	0.01271 (12)	
C10	−0.11593 (6)	0.68998 (5)	0.98022 (4)	0.01195 (12)	
C11	−0.04233 (7)	0.61895 (5)	1.03138 (5)	0.01437 (13)	
H11A	−0.0526	0.6218	1.0949	0.017*	
H11B	−0.0658	0.5625	1.0097	0.017*	
C12	0.12122 (7)	0.71385 (5)	1.06502 (5)	0.01484 (13)	
H12A	0.1987	0.7314	1.0466	0.018*	
H12B	0.1314	0.6975	1.1273	0.018*	
C13	−0.05607 (6)	0.69047 (5)	0.88921 (4)	0.01160 (12)	
C14	−0.02779 (7)	0.77725 (5)	0.85018 (4)	0.01251 (12)	
C15	−0.09918 (7)	0.84537 (5)	0.82280 (5)	0.01555 (13)	
H15A	−0.1814	0.8428	0.8269	0.019*	
C16	−0.04540 (8)	0.91968 (5)	0.78807 (5)	0.01880 (14)	
H16A	−0.0936	0.9656	0.7692	0.023*	
C17	0.07616 (8)	0.92554 (5)	0.78155 (6)	0.02024 (15)	
H17A	0.1085	0.9744	0.7573	0.024*	
C18	0.15253 (8)	0.85727 (5)	0.81173 (5)	0.01741 (14)	
C19	0.27885 (8)	0.85440 (6)	0.81185 (6)	0.02362 (17)	
H19A	0.3199	0.9005	0.7894	0.028*	
C20	0.34140 (8)	0.78384 (7)	0.84497 (7)	0.02464 (17)	
H20A	0.4241	0.7835	0.8440	0.030*	
C21	0.28357 (7)	0.71167 (6)	0.88052 (6)	0.01966 (15)	
H21A	0.3274	0.6650	0.9032	0.024*	
C22	0.16109 (7)	0.71277 (5)	0.88051 (5)	0.01407 (12)	
C23	0.07360 (6)	0.64942 (5)	0.91446 (5)	0.01248 (12)	
C24	0.09706 (7)	0.78439 (5)	0.84559 (5)	0.01361 (12)	
C25	−0.16597 (7)	0.65570 (5)	0.73850 (5)	0.01698 (14)	
H25A	−0.1511	0.6088	0.6984	0.020*	0.670 (3)
H25B	−0.1220	0.7057	0.7207	0.020*	0.670 (3)
H25C	−0.1085	0.6350	0.6992	0.020*	0.330 (3)
H25D	−0.1727	0.7168	0.7321	0.020*	0.330 (3)
C26A	−0.29771 (12)	0.67498 (9)	0.73760 (9)	0.0215 (3)	0.670 (3)
H26A	−0.3112	0.7338	0.7553	0.026*	0.670 (3)
H26B	−0.3366	0.6655	0.6788	0.026*	0.670 (3)
C26B	−0.2896 (2)	0.61090 (17)	0.71830 (17)	0.0182 (6)	0.330 (3)
H26C	−0.3382	0.6421	0.6734	0.022*	0.330 (3)
H26D	−0.2796	0.5524	0.6982	0.022*	0.330 (3)
C27	−0.34291 (9)	0.61299 (8)	0.80440 (6)	0.02674 (19)	
H27A	−0.3591	0.5573	0.7775	0.032*	0.670 (3)
H27B	−0.4152	0.6343	0.8286	0.032*	0.670 (3)
H27C	−0.3963	0.5654	0.8092	0.032*	0.330 (3)
H27D	−0.3870	0.6651	0.8107	0.032*	0.330 (3)
C28	−0.24037 (8)	0.60765 (6)	0.87702 (5)	0.01829 (14)	
H28A	−0.2329	0.5491	0.9003	0.022*	
C29	−0.24766 (7)	0.67195 (5)	0.95408 (5)	0.01499 (13)	
H29A	−0.2825	0.7249	0.9287	0.018*	
C30	−0.32410 (7)	0.64389 (6)	1.02785 (6)	0.01837 (14)	
C31	−0.41977 (9)	0.69454 (7)	1.04970 (9)	0.0308 (2)	
H31A	−0.4363	0.7452	1.0186	0.037*	
C32	−0.49117 (13)	0.67017 (9)	1.11779 (12)	0.0489 (4)	
H32A	−0.5564	0.7036	1.1306	0.059*	
C33	−0.46481 (14)	0.59586 (9)	1.16659 (11)	0.0487 (4)	
H33A	−0.5104	0.5809	1.2136	0.058*	
C34	−0.37055 (11)	0.54436 (8)	1.14494 (8)	0.0341 (2)	
H34A	−0.3530	0.4945	1.1771	0.041*	
C35	−0.30215 (8)	0.56737 (6)	1.07490 (6)	0.02335 (17)	
H35A	−0.2410	0.5314	1.0591	0.028*	
H1O2	0.0112 (16)	0.5609 (11)	0.8451 (11)	0.041 (4)*	

### Atomic displacement parameters (Å^2^)

**Table:**

	*U*^11^	*U*^22^	*U*^33^	*U*^12^	*U*^13^	*U*^23^
O1	0.0155 (2)	0.0171 (3)	0.0168 (2)	0.0037 (2)	0.00198 (18)	−0.00054 (19)
O2	0.0203 (3)	0.0111 (2)	0.0206 (2)	0.0022 (2)	0.0013 (2)	−0.00293 (19)
N1	0.0143 (3)	0.0133 (3)	0.0130 (2)	0.0012 (2)	0.00077 (19)	0.0022 (2)
N2	0.0183 (3)	0.0137 (3)	0.0118 (2)	−0.0034 (2)	−0.0016 (2)	0.0009 (2)
C1	0.0191 (3)	0.0191 (4)	0.0326 (4)	−0.0010 (3)	−0.0010 (3)	0.0005 (3)
C2	0.0227 (4)	0.0256 (4)	0.0404 (5)	−0.0068 (3)	0.0019 (4)	−0.0011 (4)
C3	0.0177 (4)	0.0324 (5)	0.0409 (5)	−0.0049 (4)	−0.0014 (3)	−0.0099 (4)
C4	0.0200 (4)	0.0298 (5)	0.0321 (4)	0.0006 (3)	−0.0086 (3)	−0.0081 (4)
C5	0.0203 (4)	0.0218 (4)	0.0217 (3)	0.0003 (3)	−0.0058 (3)	−0.0034 (3)
C6	0.0152 (3)	0.0164 (3)	0.0200 (3)	0.0005 (3)	−0.0006 (2)	−0.0036 (3)
C7	0.0147 (3)	0.0164 (3)	0.0175 (3)	0.0013 (2)	−0.0001 (2)	−0.0020 (2)
C8	0.0137 (3)	0.0159 (3)	0.0110 (2)	0.0015 (2)	0.0001 (2)	−0.0005 (2)
C9	0.0142 (3)	0.0141 (3)	0.0098 (2)	0.0008 (2)	0.0010 (2)	0.0013 (2)
C10	0.0121 (3)	0.0125 (3)	0.0114 (2)	0.0001 (2)	0.0016 (2)	0.0025 (2)
C11	0.0151 (3)	0.0142 (3)	0.0140 (3)	0.0013 (2)	0.0019 (2)	0.0043 (2)
C12	0.0151 (3)	0.0161 (3)	0.0130 (3)	0.0022 (2)	−0.0015 (2)	0.0004 (2)
C13	0.0136 (3)	0.0103 (3)	0.0109 (2)	−0.0005 (2)	0.0008 (2)	0.0011 (2)
C14	0.0163 (3)	0.0108 (3)	0.0105 (2)	−0.0006 (2)	0.0017 (2)	0.0011 (2)
C15	0.0196 (3)	0.0126 (3)	0.0145 (3)	0.0010 (3)	0.0015 (2)	0.0025 (2)
C16	0.0274 (4)	0.0122 (3)	0.0170 (3)	0.0005 (3)	0.0023 (3)	0.0032 (2)
C17	0.0283 (4)	0.0135 (3)	0.0193 (3)	−0.0046 (3)	0.0044 (3)	0.0032 (3)
C18	0.0217 (3)	0.0147 (3)	0.0163 (3)	−0.0046 (3)	0.0048 (3)	0.0007 (2)
C19	0.0218 (4)	0.0227 (4)	0.0273 (4)	−0.0073 (3)	0.0087 (3)	0.0021 (3)
C20	0.0174 (3)	0.0276 (4)	0.0299 (4)	−0.0039 (3)	0.0080 (3)	0.0006 (3)
C21	0.0154 (3)	0.0216 (4)	0.0224 (3)	0.0003 (3)	0.0048 (3)	0.0000 (3)
C22	0.0149 (3)	0.0139 (3)	0.0137 (3)	−0.0007 (2)	0.0033 (2)	−0.0005 (2)
C23	0.0139 (3)	0.0107 (3)	0.0130 (3)	0.0011 (2)	0.0017 (2)	0.0004 (2)
C24	0.0167 (3)	0.0127 (3)	0.0117 (3)	−0.0017 (2)	0.0033 (2)	0.0001 (2)
C25	0.0201 (3)	0.0179 (3)	0.0125 (3)	−0.0013 (3)	−0.0019 (2)	0.0005 (2)
C26A	0.0201 (6)	0.0247 (6)	0.0188 (5)	0.0008 (5)	−0.0056 (4)	0.0012 (4)
C26B	0.0161 (10)	0.0180 (11)	0.0195 (10)	−0.0012 (8)	−0.0047 (8)	−0.0021 (8)
C27	0.0202 (4)	0.0373 (5)	0.0221 (4)	−0.0118 (4)	−0.0034 (3)	−0.0006 (3)
C28	0.0199 (3)	0.0182 (3)	0.0166 (3)	−0.0075 (3)	−0.0004 (2)	0.0017 (3)
C29	0.0131 (3)	0.0144 (3)	0.0173 (3)	−0.0014 (2)	0.0004 (2)	0.0038 (2)
C30	0.0142 (3)	0.0175 (3)	0.0240 (3)	−0.0006 (3)	0.0051 (3)	0.0033 (3)
C31	0.0219 (4)	0.0220 (4)	0.0503 (6)	0.0045 (3)	0.0163 (4)	0.0079 (4)
C32	0.0400 (6)	0.0308 (6)	0.0813 (10)	0.0099 (5)	0.0427 (7)	0.0110 (6)
C33	0.0506 (7)	0.0319 (6)	0.0694 (9)	0.0048 (5)	0.0468 (7)	0.0116 (6)
C34	0.0356 (5)	0.0275 (5)	0.0420 (5)	0.0024 (4)	0.0236 (5)	0.0128 (4)
C35	0.0209 (4)	0.0215 (4)	0.0291 (4)	0.0019 (3)	0.0114 (3)	0.0082 (3)

### Geometric parameters (Å, °)

**Table:**

O1—C9	1.2213 (9)	C18—C19	1.4188 (13)
O2—C23	1.4056 (9)	C19—C20	1.3801 (15)
O2—H1O2	0.877 (18)	C19—H19A	0.9300
N1—C11	1.4677 (10)	C20—C21	1.4222 (13)
N1—C12	1.4737 (10)	C20—H20A	0.9300
N1—C23	1.4814 (10)	C21—C22	1.3751 (11)
N2—C13	1.4768 (10)	C21—H21A	0.9300
N2—C25	1.4800 (10)	C22—C24	1.4103 (11)
N2—C28	1.4914 (11)	C22—C23	1.5068 (11)
C1—C2	1.3901 (13)	C25—C26A	1.5083 (16)
C1—C6	1.3998 (13)	C25—C26B	1.565 (3)
C1—H1A	0.9300	C25—H25A	0.9700
C2—C3	1.3932 (16)	C25—H25B	0.9700
C2—H2A	0.9300	C25—H25C	0.9600
C3—C4	1.3917 (17)	C25—H25D	0.9600
C3—H3A	0.9300	C26A—C27	1.5068 (17)
C4—C5	1.3906 (13)	C26A—H25D	1.5542
C4—H4A	0.9300	C26A—H26A	0.9700
C5—C6	1.4022 (12)	C26A—H26B	0.9700
C5—H5A	0.9300	C26A—H27D	1.5449
C6—C7	1.4679 (11)	C26B—C27	1.463 (3)
C7—C8	1.3497 (12)	C26B—H26C	0.9700
C7—H7A	0.9300	C26B—H26D	0.9700
C8—C9	1.4982 (11)	C27—C28	1.5334 (12)
C8—C12	1.5253 (11)	C27—H27A	0.9700
C9—C10	1.5158 (11)	C27—H27B	0.9700
C10—C29	1.5299 (11)	C27—H27C	0.9600
C10—C11	1.5540 (10)	C27—H27D	0.9600
C10—C13	1.5650 (10)	C28—C29	1.5387 (12)
C11—H11A	0.9700	C28—H28A	0.9800
C11—H11B	0.9700	C29—C30	1.5125 (11)
C12—H12A	0.9700	C29—H29A	0.9800
C12—H12B	0.9700	C30—C31	1.3907 (13)
C13—C14	1.5178 (10)	C30—C35	1.4009 (12)
C13—C23	1.6117 (11)	C31—C32	1.3953 (16)
C14—C15	1.3769 (11)	C31—H31A	0.9300
C14—C24	1.4124 (11)	C32—C33	1.393 (2)
C15—C16	1.4226 (12)	C32—H32A	0.9300
C15—H15A	0.9300	C33—C34	1.3850 (17)
C16—C17	1.3779 (13)	C33—H33A	0.9300
C16—H16A	0.9300	C34—C35	1.3918 (13)
C17—C18	1.4218 (13)	C34—H34A	0.9300
C17—H17A	0.9300	C35—H35A	0.9300
C18—C24	1.4082 (11)		
			
C23—O2—H1O2	105.1 (11)	N2—C25—C26A	104.76 (7)
C11—N1—C12	108.40 (6)	N2—C25—C26B	104.28 (11)
C11—N1—C23	102.93 (6)	C26A—C25—C26B	39.77 (11)
C12—N1—C23	115.58 (6)	N2—C25—H25A	110.8
C13—N2—C25	120.12 (6)	C26A—C25—H25A	110.8
C13—N2—C28	110.27 (6)	C26B—C25—H25A	74.3
C25—N2—C28	108.73 (6)	N2—C25—H25B	110.8
C2—C1—C6	120.92 (9)	C26A—C25—H25B	110.8
C2—C1—H1A	119.5	C26B—C25—H25B	140.1
C6—C1—H1A	119.5	H25A—C25—H25B	108.9
C1—C2—C3	119.76 (10)	N2—C25—H25C	110.8
C1—C2—H2A	120.1	C26A—C25—H25C	139.6
C3—C2—H2A	120.1	C26B—C25—H25C	110.8
C4—C3—C2	119.86 (9)	H25A—C25—H25C	38.1
C4—C3—H3A	120.1	H25B—C25—H25C	74.0
C2—C3—H3A	120.1	N2—C25—H25D	110.9
C5—C4—C3	120.40 (9)	C26A—C25—H25D	74.4
C5—C4—H4A	119.8	C26B—C25—H25D	111.2
C3—C4—H4A	119.8	H25A—C25—H25D	134.7
C4—C5—C6	120.24 (9)	H25B—C25—H25D	38.2
C4—C5—H5A	119.9	H25C—C25—H25D	108.9
C6—C5—H5A	119.9	C27—C26A—C25	103.94 (9)
C1—C6—C5	118.76 (8)	C27—C26A—H25D	130.6
C1—C6—C7	118.66 (8)	C25—C26A—H25D	36.5
C5—C6—C7	122.58 (8)	C27—C26A—H26A	111.0
C8—C7—C6	127.48 (7)	C25—C26A—H26A	111.0
C8—C7—H7A	116.3	H25D—C26A—H26A	77.1
C6—C7—H7A	116.3	C27—C26A—H26B	111.0
C7—C8—C9	116.67 (7)	C25—C26A—H26B	111.0
C7—C8—C12	124.86 (7)	H25D—C26A—H26B	111.6
C9—C8—C12	118.05 (7)	H26A—C26A—H26B	109.0
O1—C9—C8	122.84 (7)	C27—C26A—H27D	36.6
O1—C9—C10	122.04 (7)	C25—C26A—H27D	131.1
C8—C9—C10	115.05 (6)	H25D—C26A—H27D	135.4
C9—C10—C29	115.01 (6)	H26A—C26A—H27D	77.2
C9—C10—C11	108.29 (6)	H26B—C26A—H27D	111.0
C29—C10—C11	117.89 (6)	C27—C26B—C25	103.27 (15)
C9—C10—C13	109.39 (6)	C27—C26B—H26C	111.1
C29—C10—C13	103.99 (6)	C25—C26B—H26C	111.1
C11—C10—C13	100.97 (6)	C27—C26B—H26D	111.1
N1—C11—C10	103.78 (6)	C25—C26B—H26D	111.1
N1—C11—H11A	111.0	H26C—C26B—H26D	109.1
C10—C11—H11A	111.0	C26B—C27—C26A	41.24 (12)
N1—C11—H11B	111.0	C26B—C27—C28	107.20 (12)
C10—C11—H11B	111.0	C26A—C27—C28	103.80 (8)
H11A—C11—H11B	109.0	C26B—C27—H27A	71.8
N1—C12—C8	115.01 (6)	C26A—C27—H27A	111.0
N1—C12—H12A	108.5	C28—C27—H27A	111.0
C8—C12—H12A	108.5	C26B—C27—H27B	137.9
N1—C12—H12B	108.5	C26A—C27—H27B	111.0
C8—C12—H12B	108.5	C28—C27—H27B	111.0
H12A—C12—H12B	107.5	H27A—C27—H27B	109.0
N2—C13—C14	116.88 (6)	C26B—C27—H27C	110.1
N2—C13—C10	104.16 (6)	C26A—C27—H27C	141.9
C14—C13—C10	117.12 (6)	C28—C27—H27C	110.3
N2—C13—C23	111.14 (6)	H27A—C27—H27C	40.5
C14—C13—C23	103.65 (6)	H27B—C27—H27C	72.2
C10—C13—C23	103.13 (5)	C26B—C27—H27D	110.5
C15—C14—C24	118.93 (7)	C26A—C27—H27D	73.8
C15—C14—C13	132.16 (7)	C28—C27—H27D	110.1
C24—C14—C13	108.89 (6)	H27A—C27—H27D	135.7
C14—C15—C16	119.05 (8)	H27B—C27—H27D	39.0
C14—C15—H15A	120.5	H27C—C27—H27D	108.5
C16—C15—H15A	120.5	N2—C28—C27	105.34 (7)
C17—C16—C15	121.83 (8)	N2—C28—C29	105.30 (6)
C17—C16—H16A	119.1	C27—C28—C29	115.11 (8)
C15—C16—H16A	119.1	N2—C28—H28A	110.3
C16—C17—C18	120.41 (7)	C27—C28—H28A	110.3
C16—C17—H17A	119.8	C29—C28—H28A	110.3
C18—C17—H17A	119.8	C30—C29—C10	116.86 (6)
C24—C18—C19	116.15 (8)	C30—C29—C28	115.17 (7)
C24—C18—C17	116.62 (8)	C10—C29—C28	102.30 (6)
C19—C18—C17	127.22 (8)	C30—C29—H29A	107.3
C20—C19—C18	120.63 (8)	C10—C29—H29A	107.3
C20—C19—H19A	119.7	C28—C29—H29A	107.3
C18—C19—H19A	119.7	C31—C30—C35	118.49 (8)
C19—C20—C21	122.20 (8)	C31—C30—C29	119.66 (8)
C19—C20—H20A	118.9	C35—C30—C29	121.85 (7)
C21—C20—H20A	118.9	C30—C31—C32	120.68 (10)
C22—C21—C20	118.18 (8)	C30—C31—H31A	119.7
C22—C21—H21A	120.9	C32—C31—H31A	119.7
C20—C21—H21A	120.9	C33—C32—C31	120.06 (10)
C21—C22—C24	119.61 (7)	C33—C32—H32A	120.0
C21—C22—C23	131.81 (7)	C31—C32—H32A	120.0
C24—C22—C23	108.56 (6)	C34—C33—C32	119.82 (10)
O2—C23—N1	108.88 (6)	C34—C33—H33A	120.1
O2—C23—C22	112.45 (6)	C32—C33—H33A	120.1
N1—C23—C22	114.82 (6)	C33—C34—C35	119.88 (10)
O2—C23—C13	109.47 (6)	C33—C34—H34A	120.1
N1—C23—C13	105.85 (5)	C35—C34—H34A	120.1
C22—C23—C13	105.00 (6)	C34—C35—C30	120.96 (9)
C18—C24—C22	123.19 (7)	C34—C35—H35A	119.5
C18—C24—C14	123.10 (7)	C30—C35—H35A	119.5
C22—C24—C14	113.68 (7)		
			
C6—C1—C2—C3	−0.56 (16)	C21—C22—C23—O2	−59.02 (11)
C1—C2—C3—C4	−1.41 (17)	C24—C22—C23—O2	122.53 (7)
C2—C3—C4—C5	1.53 (16)	C21—C22—C23—N1	66.21 (11)
C3—C4—C5—C6	0.32 (15)	C24—C22—C23—N1	−112.24 (7)
C2—C1—C6—C5	2.38 (14)	C21—C22—C23—C13	−177.98 (8)
C2—C1—C6—C7	−177.78 (9)	C24—C22—C23—C13	3.58 (7)
C4—C5—C6—C1	−2.26 (13)	N2—C13—C23—O2	0.75 (8)
C4—C5—C6—C7	177.92 (8)	C14—C13—C23—O2	−125.60 (6)
C1—C6—C7—C8	143.04 (9)	C10—C13—C23—O2	111.82 (6)
C5—C6—C7—C8	−37.13 (13)	N2—C13—C23—N1	−116.46 (6)
C6—C7—C8—C9	−174.29 (7)	C14—C13—C23—N1	117.19 (6)
C6—C7—C8—C12	−1.87 (13)	C10—C13—C23—N1	−5.38 (7)
C7—C8—C9—O1	−24.09 (10)	N2—C13—C23—C22	121.68 (6)
C12—C8—C9—O1	162.95 (7)	C14—C13—C23—C22	−4.67 (7)
C7—C8—C9—C10	153.03 (7)	C10—C13—C23—C22	−127.25 (6)
C12—C8—C9—C10	−19.93 (9)	C19—C18—C24—C22	1.84 (11)
O1—C9—C10—C29	−5.24 (10)	C17—C18—C24—C22	−177.97 (7)
C8—C9—C10—C29	177.62 (6)	C19—C18—C24—C14	179.91 (7)
O1—C9—C10—C11	−139.50 (7)	C17—C18—C24—C14	0.10 (11)
C8—C9—C10—C11	43.36 (8)	C21—C22—C24—C18	−1.44 (11)
O1—C9—C10—C13	111.30 (8)	C23—C22—C24—C18	177.23 (7)
C8—C9—C10—C13	−65.84 (7)	C21—C22—C24—C14	−179.67 (7)
C12—N1—C11—C10	74.18 (7)	C23—C22—C24—C14	−1.01 (9)
C23—N1—C11—C10	−48.73 (7)	C15—C14—C24—C18	−1.93 (11)
C9—C10—C11—N1	−70.69 (7)	C13—C14—C24—C18	179.51 (7)
C29—C10—C11—N1	156.57 (6)	C15—C14—C24—C22	176.31 (7)
C13—C10—C11—N1	44.16 (7)	C13—C14—C24—C22	−2.25 (8)
C11—N1—C12—C8	−50.47 (8)	C13—N2—C25—C26A	−109.04 (9)
C23—N1—C12—C8	64.41 (8)	C28—N2—C25—C26A	19.26 (10)
C7—C8—C12—N1	−149.86 (7)	C13—N2—C25—C26B	−150.18 (12)
C9—C8—C12—N1	22.46 (9)	C28—N2—C25—C26B	−21.88 (13)
C25—N2—C13—C14	5.38 (10)	N2—C25—C26A—C27	−34.34 (11)
C28—N2—C13—C14	−122.22 (7)	C26B—C25—C26A—C27	60.38 (17)
C25—N2—C13—C10	136.30 (7)	N2—C25—C26B—C27	32.81 (17)
C28—N2—C13—C10	8.70 (8)	C26A—C25—C26B—C27	−63.23 (16)
C25—N2—C13—C23	−113.28 (7)	C25—C26B—C27—C26A	60.14 (14)
C28—N2—C13—C23	119.12 (7)	C25—C26B—C27—C28	−31.32 (17)
C9—C10—C13—N2	−152.17 (6)	C25—C26A—C27—C26B	−64.47 (16)
C29—C10—C13—N2	−28.84 (7)	C25—C26A—C27—C28	36.01 (11)
C11—C10—C13—N2	93.80 (6)	C13—N2—C28—C27	136.76 (7)
C9—C10—C13—C14	−21.40 (8)	C25—N2—C28—C27	3.12 (9)
C29—C10—C13—C14	101.93 (7)	C13—N2—C28—C29	14.67 (8)
C11—C10—C13—C14	−135.42 (6)	C25—N2—C28—C29	−118.97 (7)
C9—C10—C13—C23	91.67 (6)	C26B—C27—C28—N2	18.50 (14)
C29—C10—C13—C23	−145.00 (6)	C26A—C27—C28—N2	−24.23 (11)
C11—C10—C13—C23	−22.35 (7)	C26B—C27—C28—C29	134.01 (13)
N2—C13—C14—C15	63.31 (10)	C26A—C27—C28—C29	91.28 (10)
C10—C13—C14—C15	−61.28 (10)	C9—C10—C29—C30	−76.41 (8)
C23—C13—C14—C15	−174.06 (8)	C11—C10—C29—C30	53.29 (9)
N2—C13—C14—C24	−118.39 (7)	C13—C10—C29—C30	164.01 (7)
C10—C13—C14—C24	117.01 (7)	C9—C10—C29—C28	156.84 (6)
C23—C13—C14—C24	4.23 (7)	C11—C10—C29—C28	−73.46 (8)
C24—C14—C15—C16	2.02 (11)	C13—C10—C29—C28	37.26 (7)
C13—C14—C15—C16	−179.82 (7)	N2—C28—C29—C30	−159.99 (6)
C14—C15—C16—C17	−0.37 (12)	C27—C28—C29—C30	84.47 (9)
C15—C16—C17—C18	−1.50 (13)	N2—C28—C29—C10	−32.16 (7)
C16—C17—C18—C24	1.59 (12)	C27—C28—C29—C10	−147.69 (7)
C16—C17—C18—C19	−178.19 (8)	C10—C29—C30—C31	117.65 (10)
C24—C18—C19—C20	−0.95 (13)	C28—C29—C30—C31	−122.23 (10)
C17—C18—C19—C20	178.83 (9)	C10—C29—C30—C35	−62.92 (11)
C18—C19—C20—C21	−0.31 (15)	C28—C29—C30—C35	57.20 (11)
C19—C20—C21—C22	0.76 (14)	C35—C30—C31—C32	0.86 (18)
C20—C21—C22—C24	0.09 (12)	C29—C30—C31—C32	−179.69 (12)
C20—C21—C22—C23	−178.21 (8)	C30—C31—C32—C33	2.0 (2)
C11—N1—C23—O2	−84.67 (7)	C31—C32—C33—C34	−2.7 (3)
C12—N1—C23—O2	157.36 (6)	C32—C33—C34—C35	0.4 (2)
C11—N1—C23—C22	148.26 (6)	C33—C34—C35—C30	2.5 (2)
C12—N1—C23—C22	30.29 (9)	C31—C30—C35—C34	−3.16 (16)
C11—N1—C23—C13	32.94 (7)	C29—C30—C35—C34	177.41 (10)
C12—N1—C23—C13	−85.03 (7)		

### Hydrogen-bond geometry (Å, °)

**Table:**

*D*—H···*A*	*D*—H	H···*A*	*D*···*A*	*D*—H···*A*
O2—H1O2···N2	0.877 (18)	1.942 (18)	2.6134 (11)	132.2 (15)
C35—H35A···O2^i^	0.93	2.54	3.3159 (13)	142

Symmetry codes: (i) −*x*, −*y*+1, −*z*+2.
